# Supporting Our Physician Parents (SOPPort): A pilot program for parental wellness at the Massachusetts General Hospital

**DOI:** 10.1017/cts.2023.645

**Published:** 2023-10-19

**Authors:** Josephine H. Li, Lauren E. Hanley, Camille E. Powe, Jacqueline A. Seiglie, Melanie S. Haines, Marc N. Wein, Amy Bregar, Karen K. Miller, Laura E. Dichtel

**Affiliations:** 1 Endocrine Division, Department of Medicine, Massachusetts General Hospital and Harvard Medical School, Boston, MA, USA; 2 Department of Obstetrics and Gynecology, Massachusetts General Hospital and Harvard Medical School, Boston, MA, USA

**Keywords:** Parental wellness, career development, mentoring, lactation support, wellness, faculty retention

## Abstract

Physician parents encounter unique challenges in balancing new parenthood with work responsibilities, especially upon their return from parental leave. We designed a pilot program that incorporated 1:1 parental coaching to expectant and new physician parents and provided stipends for lactation support and help at home. Additional initiatives included launching a virtual new parent group during the COVID-19 pandemic and starting an emergency backup pump supplies program. There was positive feedback for our Parental Wellness Program (PWP), which was used to secure expanded funding. Pilot results showed that our program had a meaningful impact on parental wellness, morale, productivity, and lactation efforts.

## Rationale for Novel Parental Wellness Program (PWP)

New physician parents and parents of young children are particularly vulnerable to burnout and reduced productivity. This is often due to child-rearing activities and lack of institutional support related to pregnancy, lactation, and parenthood [1]. Childbearing and lactating parents are disproportionately affected. The impact of new parenthood on work may contribute to the observed differences between women and men with respect to career advancement and success, including disparities in pay [2,3]. For example, female physicians in academia were identified to have an 8% lower annual salary than their male counterparts, even after adjustment for factors such as years of experience and specialty [3].

A recent nationwide survey of physician mothers identified a lack of or inconvenience of lactation facilities, lack of time available for breast pumping, discrimination because of breastfeeding, and difficulty finding childcare as prevalent negative experiences when returning from parental leave [4]. Because lactating physicians spend considerable time and effort to maintain pumping alongside clinical responsibilities, the lack of institutional support or equitable policies imposes additional stressors on an already vulnerable population [5]. Moreover, women are disproportionally burdened by a larger proportion of the mental load [6,7] and responsibilities at home [8]. The COVID-19 pandemic has exacerbated these issues and worsened existing gender disparities [9]. In particular, women experienced a higher burden of child-rearing activities during the pandemic, which have been shown to result in negative consequences on their mental health [10] and careers [11,12].

Female physicians also further experience inequities at work, including a higher burden of after-hours documentation and over 25% more patient messages compared to men [13,14]. Surveys demonstrate that female physicians are more likely to turn down academic opportunities and leadership positions and are not comfortable discussing work–life integration with department leadership [15]. Unsurprisingly, burnout rates are 30%–60% higher in women physicians versus men [16], and women physicians are more likely to leave academia [17] and/or reduce working hours [8] as compared to male physicians.

Potential solutions to these challenges have been proposed, which include the creation of mentorship programs, increasing lactation and related child-rearing support, and reduction of maternal bias in medicine [18]. However, the physician wellness landscape is lacking concrete examples of Parental Wellness Programs (PWPs) that have successfully implemented some of these proposed solutions and parental supports.

## Unmet Need

Return to work after parental leave is a stressful transition that can lead to a reduction in productivity and diminished wellness and work satisfaction. Thus, the goal of this pilot program, Supporting Our Physician Parents (SOPPort), was to (1) improve wellness, maintain productivity, and reduce burnout of expectant and new parents of all genders and gender identities; (2) provide lactation and feeding support; and (3) design a structured program that could be expanded across our institution.

## Target Audience and Funding

Interventions were designed to support junior faculty physicians who were expectant and/or new parents. The pilot program was supported by an internal Uplift Grant from the Massachusetts General (MGH) Physicians Organization (MGPO) Frigoletto Committee on Physician Well-Being. This grant supported faculty physicians of all genders and gender identities in the MGH Endocrine Division and Department of Obstetrics & Gynecology (OB/GYN) to participate in the program. Endocrine fellows were also eligible to participate through additional Endocrine Division financial support.

## Description of Interventions

The core pilot program was twofold: (1) one-on-one coaching for childbearing and non-childbearing expectant parents that provided practical advice over a maximum of four 1-hour sessions during the expectant phase through the first year of the child’s life, and (2) stipends for lactation support and help at home. We also designed and launched an emergency backup pump supplies program (MGPO Lactation Support Program) and created virtual new parent groups.

**Stipends:** Female-identifying participants and/or participants planning to lactate received a $500 lactation stipend (originally intended to defray the cost of a wearable pump but could be applied to formula or feeding supplies) and a $200 help-at-home stipend. All participants received the $200 help-at-home stipend, which was intended to cover the cost of preprepared meals, grocery delivery, emergency childcare, and/or housekeeping, with the goal of reducing stress for new parents.

**Figure 1.**
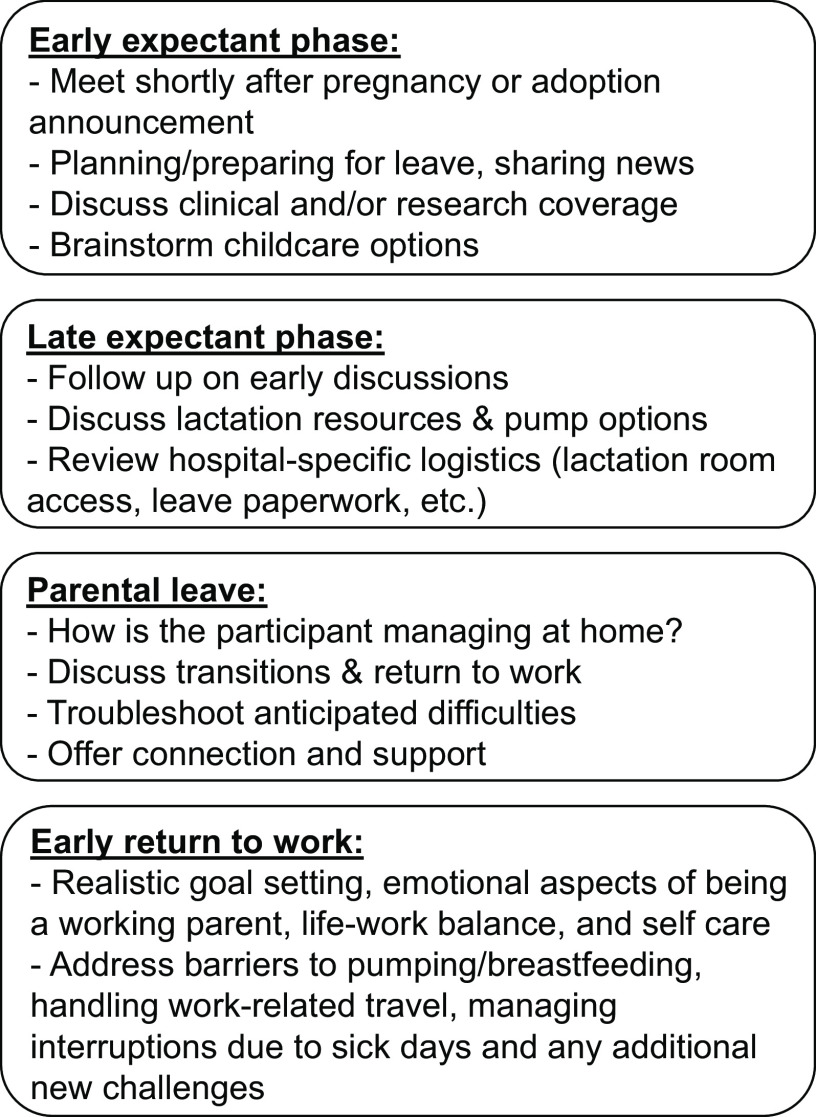
Summary of parental coaching outline.

**Coaching Program:** A coaching program for childbearing and non-childbearing expectant parents was developed with a formal outline of suggested topics to cover during each session. Each participant was matched with an “experienced” parent coach from a small pool of physician parents with recent early parenting experience. The expectation was set that discussions between the participant and coach were confidential. Coaches were expected to use the outline of topics to facilitate a session based on the individual needs of the participant. These meetings generally took place virtually due to the COVID-19 pandemic. The core outline was typically covered in four sessions for childbearing parents (Fig. 1), with two sessions during the expectant phase to prepare for leave, one session during parental leave to prepare for the return to work, and a fourth session in the 6–12 months after the child’s arrival. Nonchildbearing parents typically met twice with their coach during the program period, once before parental leave and once after. Coaches were paid $100/hour for their time, recognizing that junior faculty, particularly women, are traditionally tasked with unpaid work.

**Virtual New Parent Group:** Due to the limited social connection during the COVID-19 pandemic, a virtual new parent group was established. A group of first-time childbearing parents who delivered within a 12-month period of each other participated in this group.

**Lactation Support:** We found that lactation support was often urgently needed, particularly during the COVID-19 pandemic, and we facilitated expedited lactation support with MGH providers via phone or virtual platform. In the early pandemic, we collected and distributed position statements from professional organizations on the COVID-19 vaccine in pregnancy and lactation. A series of online pump tutorials and lactation resources were developed and hosted on a centralized website. The group additionally developed a Lactation Relative Value Unit (RVU) Reimbursement Program implemented in the Endocrine Division, which supported RVU reimbursement for one pumping slot per outpatient clinical session for a 1-year period.

In addition, we partnered with the Frigoletto Committee on Physician Well-Being to start the MGPO Lactation Support Program, which provides an emergency stock of multi-user pumps and pumping supplies (flanges, membranes, valves, tubing, bottles, and milk bags) across multiple brands that are conveniently located in a lactation room within the faculty physician lounge. Lactating individuals who inadvertently leave critical pump parts at home or encounter pump malfunctions while at work can access this room for needed items. We created a digital form with a QR code to track usage, allowing program staff to restock supplies.

**Additional Advocacy Efforts:** We revised and formalized the clinical coverage system for parental leave in the Endocrine Division to make it more equitable and avoid disproportionally burdening childbearing physicians.

## Methods of Evaluation

Since this was a novel pilot program, the evaluation goals were to gather feedback and assess the impact of the program. At the end of the pilot, we administered an anonymous survey to participants. We separately surveyed Department of Medicine (DOM) physician parents who were not in the Endocrine Division and therefore did not have the opportunity to participate in the program. For the MGPO Lactation Support Program, we conducted an anonymous survey composed of 5-point Likert-type scales and open-ended questions. The Mass General Brigham IRB confirmed that our surveys were considered program evaluation and that no IRB oversight was required. Survey questions reported in the manuscript are available in the Supplementary Appendix.

## Results

### Participants

The pilot program ran from February 2020 to March 2022. A total of 12 individuals (10 faculty and 2 fellows; all female-identifying) received lactation stipends. Fifteen individuals (11 faculty and 4 fellows; 12 female-identifying and 3 male-identifying) received help-at-home stipends. Eleven physicians were coached (7 faculty and 4 fellows; 8 female-identifying and 3 male-identifying). All faculty participants were junior faculty members at our institution, either Instructors or Assistant Professors. Anonymous feedback from participants *(n* = 6 at the time of survey) suggested that all participants (6/6, 100%) felt that the program improved their productivity upon returning to work. Parental coaching (6/6, 100%) and lactation support grants (5/6, 83%) were particularly helpful.

**Figure 2.**
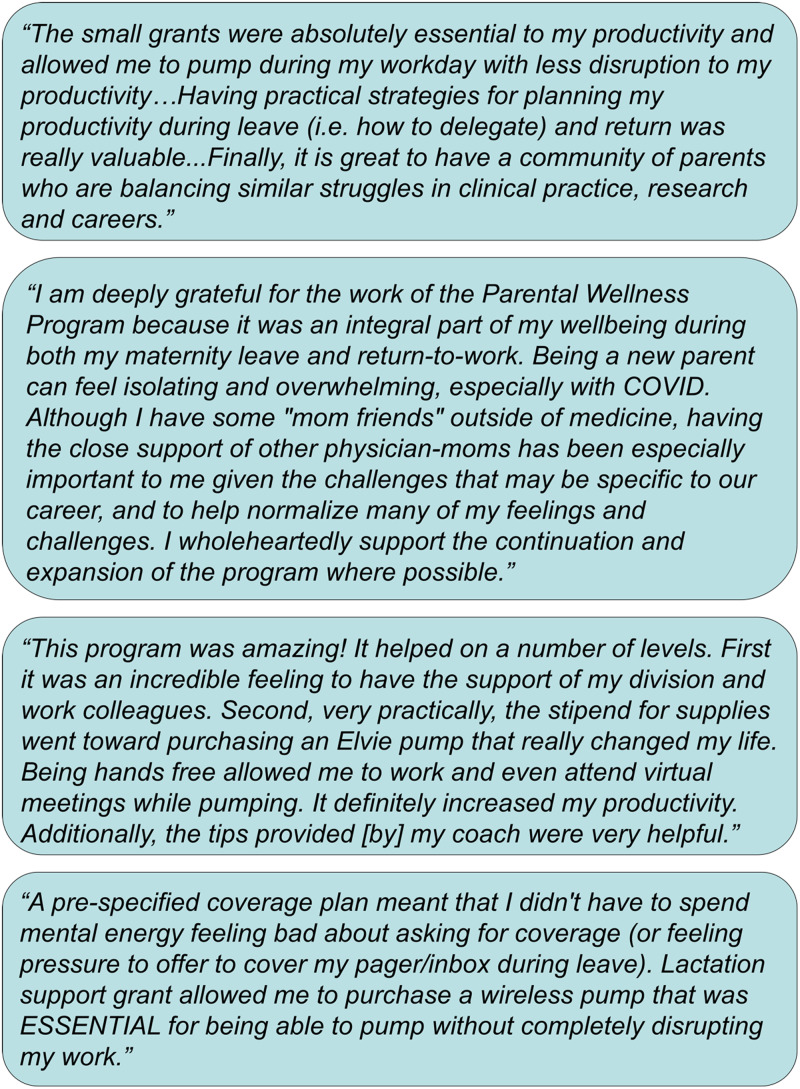
Additional testimonials from Supporting Our Physician Parents (SOPPort) participants.

One participant shared, *“I strongly believe that the program made me feel productive on my return to work during a really difficult time, made even more difficult by the uncertainties of childcare during this pandemic. I didn't have to choose between productivity and breastfeeding/pumping, and I didn't have to reinvent the wheel on return to work.”* Another participant commented, *“I cannot emphasize enough how much it meant to feel supported during this challenging time…I had my first child while involved with the program and was astounded by how hard having a baby and then returning to work can be…I am certain that this program positively impacted my mental health, work productivity, and the well-being of my family.”* Additional testimonials are shown in Figure *2*.

### Peer Group/Nonparticipants

In the DOM survey (*n* = 27 responders), 78% rated their return to work after becoming a new parent as “difficult” or “very difficult,” and 82% reported that they had nobody at work advising them on this difficult transition period. A resounding 96% of respondents stated that this pilot PWP would have been helpful to them, particularly in terms of well-being during parental leave (50%), well-being upon return to work (83%), and productivity upon return-to-work period (75%). Over 70% of respondents felt that small grants for lactation supplies (71%), individualized parental coaching (79%), and community building and connections to other new parents (79%) would have been helpful.

One faculty member reflected, *“Becoming a parent was a dramatic change for me. Of all of this, individualized coaching would have been the most helpful (especially for first time parents),”* while another emphasized, *“Just caring that people have kids, trying to create an environment where having families is welcomed and celebrated—that will speak more than anything.”*

To summarize the critical need for the program, a faculty member wrote, *“The return to work is very difficult for new parents and often times you feel isolated and afraid to voice your struggles…Support for new parents is essential. This should include acknowledgement from Division leaders on productivity goals for the first year of the child’s life. Physicians are often becoming parents while in training where financial resources to ease the stress of parents are limited. Support from MGH is essential to ensure our talented young physicians are able to remain productive and engaged physicians and parents.”*

### MGPO Lactation Support Program Evaluation

**Figure 3.**
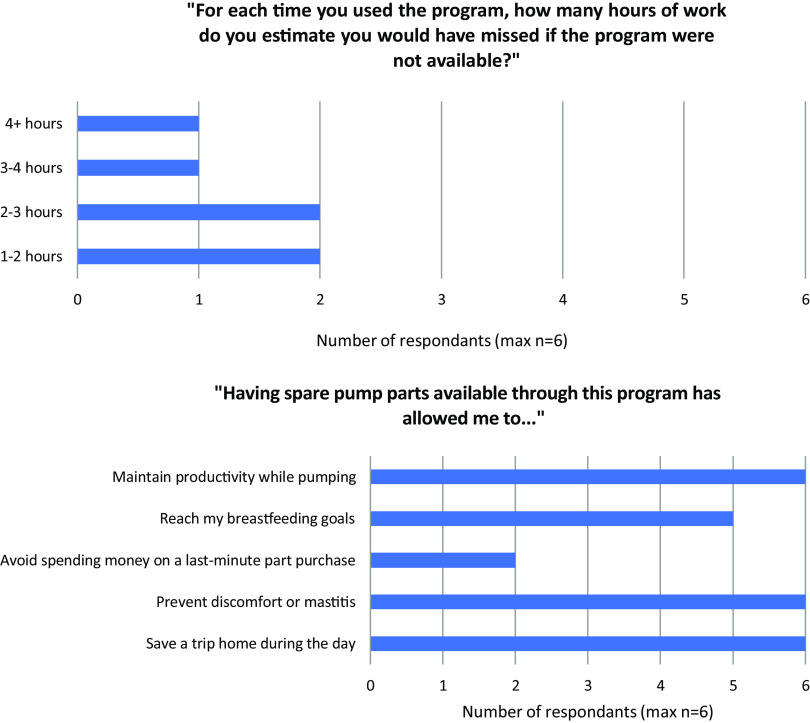
(***a***) Distribution of the number of estimated hours missed at work if the Massachusetts General Physicians Organization (MGPO) Lactation Support Program were unavailable (***b***) perceived benefits by users of the MGPO Lactation Support Program.

From June 2022 to February 2023, 10 unique individuals across multiple departments (Medicine, Surgery, and Pediatrics) utilized the program. Six individuals completed a program evaluation, of which half endorsed pumping at work to be “difficult” and 66% either agreed or strongly agreed that they were supported by the program in meeting their lactation goals. The number of hours saved due to the program was variable, ranging from 1 to 2 to more than 4 hours (Fig. 3a), and many benefits of the program were affirmed (Fig. 3b). All six participants who completed the survey strongly agreed that the program contributed to their positive health and well-being.

## Financial Cost

The MGPO Frigoletto Uplift Grant awarded a total of $23,000, which was spent across several initiatives as previously described. The maximum cost per participant was $1,100, inclusive of the $500 lactation stipend, $200 help-at-home stipend, and coaching stipend (approximately $100/hour of coaching). A separate source of funding was available to fund stipends for Endocrine Division trainee participants (see Acknowledgments). The start-up costs for supplies for the MGPO Lactation Support Program totaled approximately $3,500.

## Discussion

We established a clear and unmet need for a PWP in our survey of physician parents who did not have access to a specific parental support program. Many endorsed the lack of an advocate to help navigate the transition back to work, the isolation of new parenthood, challenges of reduced productivity, and the importance of a supportive environment. In addition, there was overwhelming positive feedback from participants of the formalized PWP indicating that the program directly addressed some of these issues.

Participant feedback specifically highlighted that the program enhanced well-being, promoted productivity, helped avoid “reinventing the wheel,” normalized the challenges of early parenting, increased perceptions of institutional support, and supported lactation efforts. This was consistent with previously published programs targeting breastfeeding support, of which one showed that having access to a wearable breast pump was associated with lactating physicians’ ability to meet their breastfeeding goals and take shorter lactation breaks [19]. We also supported Endocrine Division clinical trainees, who have even less control over their schedules and are more vulnerable to work-related pressures than attending physicians [20].

We believe that this unique coaching model was particularly effective for multiple reasons: (1) coaches were experienced and knowledgeable individuals who had recently been new faculty parents themselves at our institution; (2) the coaching session outline targeted critical issues related to parental leave, lactation, and return to work that participants would not have otherwise anticipated; (3) coaching sessions permitted a safe space to discuss parental wellness-related concerns; and (4) participants had a supportive coach and advocate to help them during a truly vulnerable time. A limitation of this brief report is the lack of use of validated surveys, which are generally unavailable in the parental wellness space at this time. However, we believe that it is critical to report these data due to the lack of published experience with successful PWPs for physician parents.

The cost of implementing the pilot program was relatively modest. This PWP was subsequently funded by a larger MGPO Phoenix Wellness Grant and was upscaled to serve all faculty in the MGH DOM. The program was smoothly expanded to this larger audience of nearly 70 participants (83% female), and it again received very positive testimonial feedback with formal surveys nearing completion. The growth and expansion of the program required physician leadership, administrative support, and organizational buy-in. It was critical to have physician leaders in the PWP who acted as champions for new parents, responded to feedback, established a sense of community, and developed new programming to meet the needs of physician parents.

The authors hope to continue this important parental wellness work with an emphasis on supporting junior faculty parents in medicine and promoting a wider change in culture. Through these efforts, we hope to improve parental wellness, morale, and satisfaction, reduce burnout at a particularly vulnerable time, reduce gender disparities, and promote community building among parents of young children.

## Supporting information

Li et al. supplementary material Li et al. supplementary material

## Supplementary material

The supplementary material for this article can be found at https://doi.org/10.1017/cts.2023.645.
