# Supplementary material for: Supporting Our Physician Parents (SOPPort): A pilot program for parental wellness at the Massachusetts General Hospital
Source: J Clin Transl Sci. 2023 Oct 19;7(1):e238. doi: 10.1017/cts.2023.645 (PMC10663770; doi:10.1017/cts.2023.645)
Supplement: Li et al. supplementary material [file S2059866123006453sup001.docx]

**Supplemental Appendix: Reported Survey Questions**

1. Participant Survey

*Do you think the Program improved your productivity on return to work?*

*(Response options: Yes/No)*

*How much did the following aspects of the program help:*

|  | *Not at all* | *Somewhat* | *A lot* | *Not applicable (did not experience)* |
| --- | --- | --- | --- | --- |
| *Program in general* |  |  |  |  |
| *Coaching with a focus on practical strategies* |  |  |  |  |
| *Small grants for lactation/support and help at home* |  |  |  |  |
| *Community building/connections to other new parents* |  |  |  |  |

*Please provide any comments or suggestions you may have on these issues or other parental wellness initiatives.*

*(Response option: Free text)*

1. Non-participant Survey

*How difficult was your transition back to work after becoming a new parent?*

*(Response options: Very difficult, difficult, neutral, fairly easy)*

*Was there someone at work who provided help with your transition back to work?*

*(Response options: Yes/No)*

*Do you think that the Parental Wellness Program (see email description and brochure attached to this email) would have helped you?*

*(Response options: Yes/No/NA)*

*What would it [the Parental Wellness Program] have helped (check all that apply):*

1. *My wellbeing during parental leave*
2. *My wellbeing on return to work*
3. *My productivity on return to work*
4. *Other*

*Which of the following services [from the Parental Wellness Program] would have helped?*

1. *Coaching with a focus on practical strategies*
2. *Small grants for lactation/support and help at home*
3. *Community building/connections to other new parents*

*Please provide any comments or suggestions you may have on these issues or other parental wellness initiatives.*

*(Response option: Free text)*
